# Galectin-6 is a novel skin anti-microbial peptide that is modulated by the skin barrier and microbiome^⿿^

**DOI:** 10.1016/j.jdermsci.2016.06.008

**Published:** 2016-10

**Authors:** Ken Natsuga, -->Fiona M. Watt

**Affiliations:** Department of Dermatology, Hokkaido University Graduate School of Medicine N15W7, Sapporo 060-8638, Japan; Centre for Stem Cells and Regenerative Medicine, King⿿s College London, 28th floor, Guy⿿s Tower Wing, London SE1 9RT, UK

**Keywords:** EPI⿿/⿿, lacking Envoplakin, Periplakin and Involucrin, AMPs, anti-microbial proteins, WT, wild type, SPF, specific pathogen-free, GST, Glutathione S-transferase, Epidermal barrier, Galectin, Antimicrobial peptides, Envoplakin, Periplakin, Involucrin

To the Editor,

We have previously reported that mice lacking three components of the epidermal cornified envelope ⿿ Envoplakin, Periplakin and Involucrin ⿿ (EPI⿿/⿿ mice) exhibit several hallmarks of atopic dermatitis and are resistant to developing chemically induced skin tumours [1], [2]. EPI⿿/⿿ mice have an enhanced skin bacterial load and exhibit upregulation of a variety of anti-microbial proteins (AMPs), including β-defensins and S100A9 [3].

When analysing a previously published dataset of genes that are differently expressed in the skin of EPI⿿/⿿ and wild type (WT) mice, we noticed a more than 3-fold increase in expression of Galectin-6 [2]. Galectins are a family of lectins with binding specificity for β- galactosides [4]. Although the function of Galectin-6 has not been characterized, several galectin family members have antimicrobial activity, targeting blood group positive microbes [5]. This led us to hypothesise that Galectin-6 is a novel skin AMP.

When EPI⿿/⿿ mice are housed under conventional conditions, the skin microbiota, as quantitated by 16 S ribosomal RNA levels, is approximately three-fold more abundant than in WT controls [3] (see Supplementary Information for details of mice and experimental techniques). However, when EPI⿿/⿿ mice are housed under specific pathogen-free (SPF) conditions the bacterial load falls to that of WT mice [3]. We confirmed that Galectin-6 mRNA was significantly higher in EPI⿿/⿿ mice (Fig. 1A). Furthermore, in contrast to the AMPs analysed previously [3], Galectin-6 expression was decreased in the skin of EPI⿿/⿿ mice housed under SPF conditions (Fig. 1A).

**Fig. 1:**
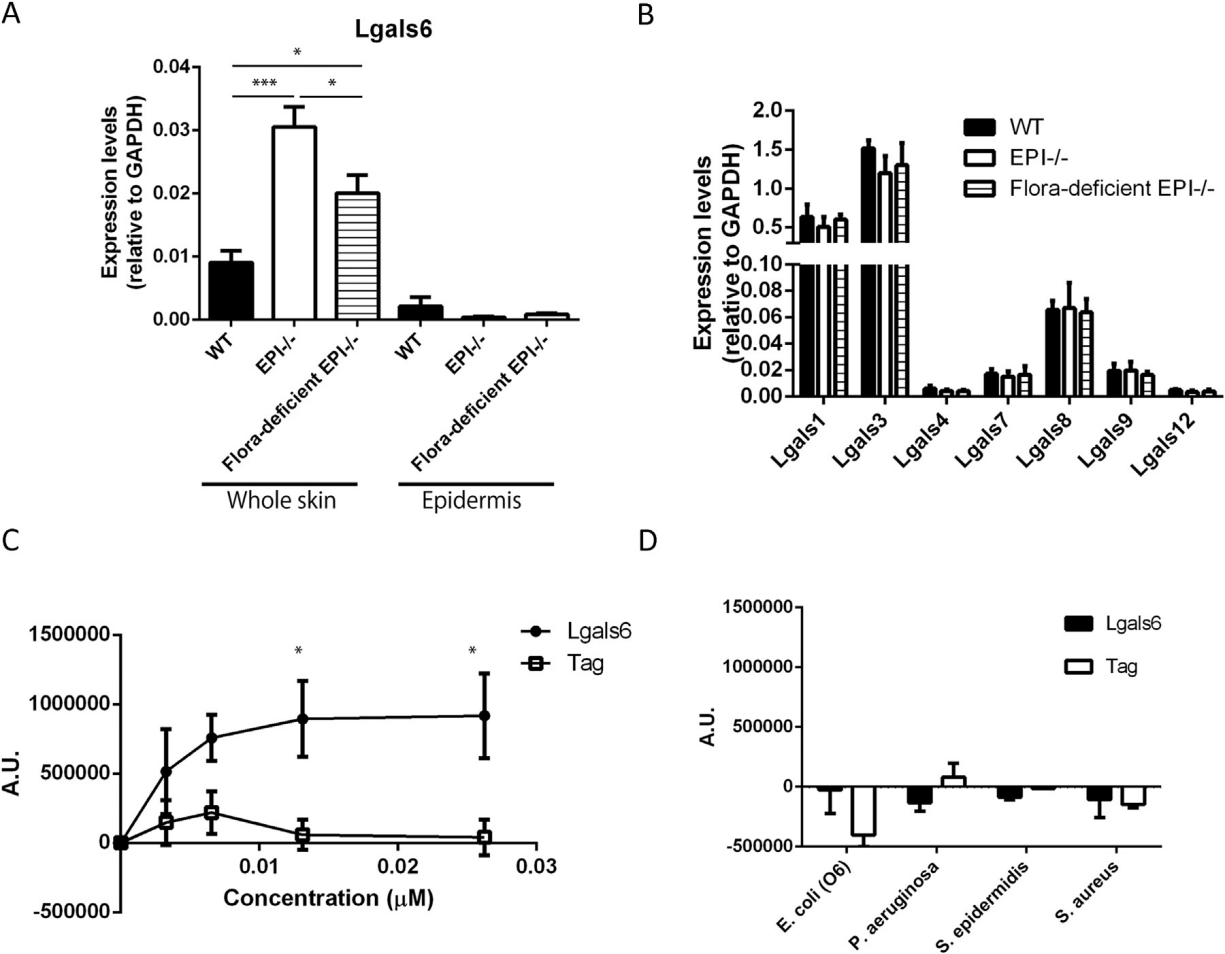
Lgals6 expression and function in skin (A) qRT-PCR of Lgals6 in WT, EPI⿿/⿿ and flora-deficient EPI⿿/⿿ skin and epidermis. (B) qRT-PCR of genes encoding other galectins (Lgals1, Lgals3, Lgals4, Lgals7, Lgals8, Lgals9 and Lgals12) in WT, EPI⿿/⿿ and flora-deficient EPI⿿/⿿ whole skin. Data are means ± SEM from at least 4 mice per group. (C) Antimicrobial properties of recombinant galectin-6 (closed circles) or tag protein (open squares) on blood group antigen-expressing E.coli (O86) growth. (D) Growth of non-blood group antigen-expressing E.coli (O6), P. aeruginosa, S. epidermidis and S. aureus treated with the recombinant galectin-6 or tag protein. See supplemental information for methodology and reagents. P-values are indicated with: * p < 0.05, *** p <0.001.

By comparing levels of Galectin-6 in total skin, from which the adipocyte layer had been removed, and epidermis, we could show that Galectin-6 is more abundant in the dermis than the epidermis (Fig. 1A). This is of interest, given that bacteria can penetrate the living epidermal layers in EPI⿿/⿿ mice [3]. Since galectin-6 gene expression in WT skin was not altered when WT mice were housed under SPF conditions (**Supplementary** Fig. 1), galetin-6 is likely to respond to an abundant skin flora when the epidermal barrier is defective, but not to microbial load under steady state conditions.

Amongst galectin family members, galectin-4 shares more than 80% sequence homology with galectin-6. Lgals6 represents a duplication of the galectin-4 gene, and the two genes share 8 out of 10 exons [6]. However, expression of other galectins, including galectin-4, did not differ between EPI⿿/⿿ and WT whole skin, irrespective of whether or not the EPI⿿/⿿ mice were maintained under SPF conditions (Fig. 1B).

Given its unusual expression in EPI⿿/⿿ skin, we investigated whether Galectin-6 has anti-microbial activity. A range of bacterial strains were grown in the presence of recombinant Galectin-6 or control protein expressing GST tag only. As previously reported for Galectin-4 and Galectin-8 [7], Galectin-6 suppressed the growth of E. coli expressing blood group antigen (*E. coli* O86, possessing blood group-B antigen), in a dose-dependent manner (Fig. 1C), and did not affect the growth of non-blood group antigen-expressing E.coli or other bacterial strains tested (Fig. 1D). The antimicrobial activity of galectin-6 was similar to that of murine galectin-4 (Supplementary Fig. 2). We conclude that Galectin-6 is a novel AMP, the expression of which can be modulated by skin bacterial load.

Galectin-6 differs from the other AMPs we have examined in being regulated by bacterial load in EPI⿿/⿿ skin. This is all the more remarkable, given that its abundance is low in the epidermis relative to total skin. Lgals6 is also unusual in that it does not have a human homologue and is present in some mouse species, including 129sv, but not in others, such as C57BL/6 and Balb/c [6]. Even though Galectin-4 and Galectin-6 are highly conserved, their expression patterns in the digestive tract differ following challenge with dextran sodium sulfate [8]. This is consistent with the differential regulation of Lgals6 and Lgals4 by the skin microbiota. Furthermore, previously published microarray data show that Lgals4 expression is not significantly altered in atopic dermatitis and psoriatic skin compared with normal controls (GSE26952) [9]. We cannot exclude the possibility that skin barrier defects also contributed to the upregulated expression of Lgals6 in EPI⿿/⿿ mice, because the level in flora-deficient EPI⿿/⿿mice was still higher than that of controls. Nevertheless, our findings reveal that Galectin-6, unlike other AMPs [3], is influenced by the abundance of skin microbiota. We also do not rule out additional functions for Galectin-6 in the skin; for example in the dermis it could potentially modulate fibrosis, as in the case of galectin-3 in the lung [10].

## Conflict of interest

The authors state no conflict of interest.
