# Supplementary material for: Galectin-6 is a novel skin anti-microbial peptide that is modulated by the skin barrier and microbiome
Source: J Dermatol Sci. 2016 Oct;84(1):97–9. doi: 10.1016/j.jdermsci.2016.06.008 (PMC5104689; doi:10.1016/j.jdermsci.2016.06.008)
Supplement: Supplementary file 1 [file mmc1.docx]

**Supplementary Material and Methods**

**Mice**

Mice deficient for Ppl/Evpl/Ivl (EPI-/-) mice were generated as reported previously [[1](#_ENREF_1)]. The genetic background of EPI-/- mice is composed of Sv129 (40.98%, ± 1.52), C57BL/6 (51.39%, ± 1.62) and BALB/c (4.7%, ± 1.24) [[2](#_ENREF_2)]. Accordingly, the F2 generation of crosses between Sv129 and C57BL/6 mice was used as the WT control (51.82%, ± 3.35 Sv129; 49.92%, ± 1.98 C57BL/6) as described previously [[2](#_ENREF_2)]. Mice, unless otherwise specified, were kept in a conventional facility. For depletion of skin microbiota, mice were kept in an SPF barrier facility. Entry into the SPF facility required staff to change into scrubs and protective footwear, to take an air shower and use hand disinfectant [[3](#_ENREF_3)].

**Quantitative PCR (qPCR)**

To assess galectin expression in skin, dorsal skin of mice devoid of subcutaneous fat was harvested and was used as the whole skin sample. To obtain epidermal samples, whole skin specimens were incubated in PBS at 56^0^C for 30 seconds and scraped with a scalpel to collect the epidermis. Whole skin or epidermal samples were processed with a Precellys 24 homogenizer (Bertin Technologies, France) and RNA was extracted using RNeasy (Qiagen, UK). cDNA was synthesized with SuperScript III (Life Technologies, NY). qRT-PCR was performed using SYBR Green and designed primers and was run on an ABI7000. Gapdh was used as a housekeeping control gene. The primers used were as follows. Lgals1: CAAAGTTCGGGGAGAGGTGG (forward), TGGGCATTGAAGCGAGGATT (reverse); Lgals3: CACAGTGAAACCCAACGCAA (forward), AGCGGGGGTTAAAGTGGAAG (reverse); Lgals4: ATGGTCACCCATCTGCAAGT (forward), AGCTGGAATAGTCATGGCTCC (reverse); Lgals6: ACGAAGCAAAGCGGACGATGGG (forward), CGGTGCCCGTATTCATAGAAGGGA (reverse); Lgals7 AGCTGAACCACTACCTTGCC (forward), GTGCTGGGTAGCAGACATGG (reverse); Lgals8: TTGAATGCCTCCATGGGTCC (forward), GCTTTCACATTGAGGCGTGG (reverse); Lgals9: GATGGCTCTCTTCAGTGCCC (forward), CAGCCCTCCTTGGATTGGTC (reverse); Lgals12: TCCCACTGTCACGGGTAGAT (forward), GGGCTATACAGCAGGAAGGG (reverse); Gapdh: AACATCAAATGGGGTGAGGCC (forward), GTTGTCATGGATGACCTTGGC (reverse).

To confirm reduced bacterial load in mice skin kept under SPF conditions, 5mm diameter skin biopsies were collected from the ears of age- and sex-matched mice using sterile instruments. DNA was extracted with the PureLink Genome DNA Mini Kit (Life Technologies) following a Precellys 24 bead-beating step. 16S rRNA gene qPCR was performed as described above using the following primers (Bact-8F: AGAGTTTGATCCTGGCTCAG, Bact-338R: CTGCTGCCTCCCGTAGGAGT) [[3](#_ENREF_3)].

**Antimicrobial assay**

E. coli (O6, ATCC25922), P. aeruginosa (ATCC27853), S. aureus (ATCC29213), S. epidermidis (ATCC12228) and BGB+ E. coli (ATCC 12701) were grown in LB broth at 37^0^C overnight. The overnight culture was diluted 100-fold in LB broth and used as an inoculum. The inoculum was incubated with recombinant Galectin-6 protein, murine Galectin-4 protein (yeast derived GST-tagged protein, CUSABIO, China) or GST-tag protein or buffer at 37^0^C for 2 hours. The BacTiter-Glo Microbial Cell Viabitliy Assay (Promega, WI) was used to monitor bacterial growth. Luminsescence was measured using LAS-4000 (FujiFilm, Japan) and MultiGauge v3.1 software. Antimicrobial properties (A.U.) were calculated as (luminescence of buffer-treated bacteria) minus (luminescence of galectin-6 or tag-treated bacteria). For production of GST-tag protein, pGEX-6P1 (GE Healthcare, WI) was transformed into BL21 E.coli grown in LB broth. Protein expression was obtained through the Overnight Express Autoinduction System (Novagen, Germany). GST-tag protein was purified using GSTrap column (GE Healthcare).

**References**

[1] Sevilla LM, Nachat R, Groot KR, Klement JF, Uitto J, Djian P, et al.: Mice deficient in involucrin, envoplakin, and periplakin have a defective epidermal barrier. The Journal of cell biology 179: 1599-1612, 2007.

[2] Cipolat S, Hoste E, Natsuga K, Quist S, Watt FM: Epidermal barrier defects link atopic dermatitis with altered skin cancer susceptibility. Elife 3: e01888, 2014.

[3] Natsuga K, Cipolat S, Watt FM: Increased Bacterial Load and Expression of Antimicrobial Peptides in Skin of Barrier-Deficient Mice with Reduced Cancer Susceptibility. The Journal of investigative dermatology 136: 99-106, 2016.

**Supplementary Figure 1. Lgals6 expression in flora-deficient WT mice**

qRT-PCR of Lgals6 in WT and flora-deficient WT skin (A) and epidermis (B). Data are means ± SEM from 5 mice per group.

(A) Whole skin





(B) Epidermis





**Supplementary Figure 2. Antimicrobial properties of recombinant murine galectin-4 on blood group antigen-expressing E.coli (O86) growth**
